# Comparison of internal medicine applicant and resident characteristics with performance on ACGME milestones

**DOI:** 10.1080/10872981.2023.2211359

**Published:** 2023-05-11

**Authors:** Alex G. Ryden, Trent C. Fuller, Richard S. Rose, Katie L. Lappe, Sonja Raaum, Stacy A. Johnson

**Affiliations:** a Internal Medicine Residency Program, University of Utah School of Medicine, Salt Lake City, UT, USA; b Division of General Internal Medicine, University of Utah School of Medicine, Salt Lake City, UT, USA; c Department of General Internal Medicine, Salt Lake City Veterans Affairs Medical Center, Salt Lake City, UT, USA

**Keywords:** Resident, performance, ACGME milestones, prediction, internal medicine

## Abstract

Internal medicine (IM) residency programs select applicants based on several metrics. Factors predicting success during residency are unclear across studies. To identify whether specific applicant or resident factors are associated with IM resident performance using ACGME milestones. We tested for associations between applicant factors available prior to the start of IM residency and resident factors measured during IM residency training, and resident performance on ACGME milestones across three consecutive years of IM training between 2015-2020. Univariable and multivariable linear regression modeling was used to test associations. Eighty-nine categorical IM residents that completed 3 consecutive years of training were included. Median age was 28 years (IQR 27-29) and 59.6% were male. Mean ACGME milestone scores increased with each post-graduate year (PGY) from 3.36 (SD 0.19) for PGY-1, to 3.80 (SD 0.15) for PGY-2, to 4.14 (SD 0.15) for PGY-3. Univariable modeling suggested referral to the clinical competency committee (CCC) for professionalism concerns was negatively associated with resident performance during each PGY. No applicant or resident factors included in the final multivariable regression models (age at starting residency, USMLE Step scores, interview score, rank list position, ITE scores) were associated with ACGME milestone scores for PGY-1 and PGY-2. Referral to the CCC for professionalism was negatively associated with resident performance during PGY-3. Residency selection factors did not predict resident milestone evaluation scores. Referral to the CCC was associated with significantly worse resident evaluation scores, suggesting professionalism may correlate with clinical performance.

## Introduction

Residency programs rely on several metrics to screen and rank applicants for the National Residency Match Program [1]. Objective measures such as the United States Medical Licensing Examination (USMLE) Step scores are frequently used [2], however the correlation between Step scores and residency performance is unclear [3,4].

Factors during residency may also affect performance and it is unclear which factors could contribute to residency performance. While several factors are reported to correlate with resident performance, differences in study design, tested characteristics, medical/surgical specialty, and definition of success limit the generalizability of these findings across residency programs [3,5,6]. The lack of standardized evaluation of residents represents a notable gap, with prior studies relying on different evaluation methods [5,7,8].

In July 2013, the Accreditation Council for Graduate Medical Education (ACGME) launched a milestone-based evaluation, which was developed as a standardized framework to assess competency across key professional domains, and to create a logical trajectory of resident performance [9]. Recently, the relationship between internal medicine (IM) residency applicant characteristics and intern year performance on ACGME milestones reported by Golden, *et al* suggest applicant characteristics were not reflective of performance [10]. Considering that resident knowledge and responsibilities typically increase over time, it is possible unique resident characteristics are more predictive of resident performance than applicant factors. To date, no studies have assessed how applicant and resident characteristics longitudinally affect residency performance.

In this study, we aimed to identify applicant and resident factors associated with IM residency performance as measured by ACGME milestones during each post-graduate year. We hypothesized that a longitudinal assessment of resident performance over several years may identify characteristics predictive of performance during IM residency.

## Methods

### Study design and setting

We performed a predictive modeling study at the University of Utah IM Residency Program, an ACGME accredited site. Residents primarily rotate between three hospital-based settings: 1) University of Utah Medical Center, a 548-bed academic medical center; 2) Intermountain Medical Center, a 452-bed community hospital; and 3) George E. Wahlen Department of Veterans Affairs Medical Center, a 127-bed level 1a facility. The study period began 1 July 2015, and ended 1 July 2020.

### Participants

Categorical IM and combined medicine/pediatric residents who completed at least three consecutive post-graduate years (PGY) of training during the study period were included. For categorical IM residents that were selected to complete an additional chief medical resident (CMR) year, the CMR year was excluded from analysis. Similarly, for combined medicine/pediatric residents, only IM clinical rotations were included. Preliminary interns were excluded as we aimed to evaluate residents longitudinally.

### Predictive factors and study outcomes

Several factors were identified as potential predictors of resident clinical performance including applicant factors such as age when starting residency, gender identity, USMLE Step 1 and Step 2 Clinical Knowledge (CK) scores, position on rank list for residency match, and residency interview score as well as other factors that occurred during residency training such as In-Training Examination (ITE) scores, Step 3 score, and referral to the Clinical Competency Committee (CCC) for discussion of professionalism concerns. These factors were chosen as prior studies have attempted to evaluate the correlation with these factors to performance (but have not previously been studied in a longitudinal fashion over an entire Internal Medicine Residency). Interview scores are unique to our program and we wanted to study any correlation with these scores to performance. The impact of age and gender on performance is unclear but we included these factors as they had not been studied at our institution.

The CCC is a forum for evaluating resident performance in any of the ACGME six core competencies. At our institution, the CCC is tasked to identify residents who are not making expected progress and design individualized learning plans. We excluded residents referred to the CCC solely for medical knowledge issues, as medical knowledge was assessed independently in our study by ITE scores. Only residents with professionalism concerns were included in the ‘referral to CCC for professionalism’ variable as these made up the majority (86%) of referrals after excluding medical knowledge deficiencies. Professionalism concerns were identified from evaluation comments and were not derived from the ACGME Professionalism core competency domain.

Interview scores at our program are assigned to an applicant by each of two interviewers. Scores range from 1 to 5 with 5 being a highly desirable candidate. Interviewers have the entire Electronic Residency Application System (ERAS) application prior to the interview and the score is a subjective rating based on a holistic review of the applicant. All interviewers are given a scoring guide when rating applicants. The main outcome variables were the mean ACGME milestone score achieved at the completion of each PGY (i.e., PGY-1, PGY-2, and PGY-3). Mean milestone scores were derived from attending performance evaluations that are completed on each resident at the end of their rotation. The performance evaluations were designed to identify and rate the individual ACGME milestone core competency domains: Patient Care and Procedural Skills, Medical Knowledge, Systems-Based Practice, Practice-Based Learning and Improvement, Professionalism, Interpersonal and Communication Skills. The mean milestone score was calculated from a compilation of the core competency domain scores. Milestone scores were aggregated at the end of each residency year. This method was felt to attenuate differences in assessors and potentially differences between how different clinical rotations may be assessed.

### Data sources

Data extracted from residency program records included age when starting residency, gender, USMLE Step 1, Step 2-CK, and Step 3 scores, residency rank list position, and ITE scores. The outcome variables were extracted from two different online databases (E-value 2015–2017 and MedHub 2017–2020) used by the residency program to track resident clinical performance evaluations during the study period. The reason for referral to the CCC was extracted from a manual review of monthly CCC meeting minutes.

### Statistical analysis

Baseline characteristics are reported as frequency and percent for categorical variables and median with interquartile range (IQR) for continuous variables. Due to the perceived sensitivity of USMLE Step and ITE data, scores are reported as normalized values. The 2017 mean USMLE Steps 1, 2 CK, and 3 scores were used as the normalizing factor for the 25^th^ and 75^th^ percentile and median for each corresponding Step score. The median ITE score for PGY-1 (ITE 1) was used as the normalizing factor for the 25^th^ and 75^th^ percentile and median values for each ITE (*i.e*., ITE 1, ITE 2, and ITE 3). Univariable and multivariable regression models were fitted for each PGY mean milestone score from PGY-1 to PGY-3 based on prior research and content expertise. Covariates included in the final regression models were: age when starting residency; USMLE Step 1, 2 CK, and 3 scores; rank list position (as a continuous variable); average interview score; ITE scores up to current PGY level (e.g., ITE 1 and ITE 2 were included in model for PGY-2 milestones, and ITE 3 was excluded as the ITE 3 had not been completed at the end of PGY-2); and referral to CCC. Given the multiple comparisons of predictor variables with outcomes stratified by PGY, a Bonferroni correction was applied to provide a more conservative *p*-value threshold (<0.017) for significance [11]. Based on the results from our primary analysis, an exploratory, post-hoc sensitivity analysis was performed to test for associations between individual ACGME core competencies and referral to the CCC. Stata/IC version 16.1 (StataCorp, College Station, TX) was used for all analyses. The local institutional review board deemed this study exempt (IRB_00127307).

## Results

A total of 89 individuals completed three consecutive years of IM residency training during the study period and were included. Two residents in the physician scientist training program advanced to fellowship after completion of PGY 2 and, therefore, are not included in the analysis for PGY 3. The median age at the start of residency was 28 years (IQR 27–29) and the majority were male (59.6%). The normalized median Step 1, Step 2 CK and Step 3 scores were 1.03 (IQR 0.98–1.09), 1.01 (0.98–1.05) and 1.03 (0.99–1.07) respectively. The mean ACGME milestone scores increased with each year of PGY training from 3.36 (SD 0.19) for PGY 1, to 3.80 (SD 0.15) for PGY 2, and 4.14 (SD 0.15) for PGY 3 (Table 1). Normalized median ITE scores also increased for each PGY year of training, as shown in Table 1.

**Table 1.** Baseline characteristics of categorical internal medicine residents for academic years 2015/2016 through 2017/2018.

Baseline Characteristic	Categorical Internal Medicine Residents for Academic Years 15/16–17/18
Total Residents, n (%)	89 (100)
Male, n (%)	53 (59.6)
Age in years when starting residency, median (IQR)	28 (27–29)
Rank list position, median (IQR)	116 (70–155)
Interview Score, median (IQR)	4 (3.5–4.5)
Normalized Median USMLE Scores^a^	
*Step 1, median (IQR)*	1.03 (0.98–1.09)
*Step 2 CK, median (IQR)*	1.01 (0.98–1.05)
*Step 3, median (IQR)*	1.03 (0.99–1.07)
Average ACGME Aggregate Milestone Score by PGY	
*PGY 1, mean (SD)*	3.36 (0.19)
*PGY 2, mean (SD)*	3.80 (0.15)
*PGY 3, mean (SD)*	4.14 (0.15)
Normalized Median In-Training Exam Percentile^b^	
*ITE1, median (IQR)*	1.00 (0.60–1.24)
*ITE2, median (IQR)*	1.04 (0.71–1.17)
*ITE3, median (IQR)*	1.07 (0.77–1.26)
Referral to Clinical Competency Committee, n (%)	13 (14.6)

Abbreviations: IQR- interquartile range; USMLE – United States Medical Licensing Examination; ACGME – American College of Graduate Medical Education; PGY – Post Graduate Year; SD – standard deviation; ITE – In-Training Exam.

Footnotes: a) 2017 USMLE mean scores were used as the normalizing values for the corresponding 25th and 75th quartiles and median values of Step 1, Step 2 CK, and Step 3 scores; b) ITE1 median percentile was used as the normalizing value for all 25th and 75th quartiles and median ITE2 and ITE3%iles.

### Univariable regression analysis

Results of the univariable analysis are presented in Table 2. Specifically, USMLE Step 3 scores were associated with small, significant increases in mean milestone scores during each PGY of training (beta coefficients ranged from 0.005–0.01, *p* = 0.001). Step 2 CK scores also appeared to be weakly associated with milestone scores during PGY-2 and PGY-3 (Table 2). Similarly, ITE scores available during the current year of residency (e.g., ITE 2 during PGY-2) and preceding years of PGY training (e.g., ITE 2 during PGY-3) were associated with small, significant increases in mean milestones (beta coefficients ranged from 0.002–0.003). Significantly lower mean milestone scores during each year of residency were observed among residents referred to the CCC due to professionalism issues (beta coefficients ranged from −0.12 to −0.21). Applicant factors including age at the start of residency, gender, average interview score, and rank list position were not significantly associated with milestone scores, except during PGY-2 where a small negative association was noted between rank list and mean milestone score (beta = −0.001 [95% CI −0.001, −0.0001], *p* = 0.013).

**Table 2.** Univariable analysis of predictors of aggregate ACGME milestone scores by post-graduate year of internal medicine residency.

Predictor of Average Milestone Score	PGY 1		PGY 2		PGY 3	
	β (95% CI)	*P*-value*	β (95% CI)	*P*-value*	β (95% CI)	*P*-value*
Age at start of residency	−0.01 (−0.03, −0.00)	0.042	−0.001 (−0.01, 0.01)	0.861	−0.01 (−0.02, 0.002)	0.092
Male gender	−0.01 (−0.09, 0.07)	0.811	0.01 (−0.06, 0.07)	0.874	−0.012 (−0.08, 0.05)	0.717
Interview Score	0.05 (−0.02, 0.11)	0.186	0.05 (−0.0002, 0.11)	0.051	0.07 (0.01, 0.13)	0.019
Rank list position	−0.0001 (−0.001, 0.001)	0.726	−0.001 (−0.001, −0.0001)	**0.013**	−0.0004 (−0.001, 0.0001)	0.120
USMLE Step Score						
*Step 1*	0.002 (−0.001, 0.004)	0.197	0.003 (0.001, 0.01)	**0.002**	0.003 (0.0004, 0.005)	0.021
*Step 2*	0.003 (−.0003, 0.01)	0.074	0.003 (0.001, 0.01)	**0.005**	0.004 (0.002, 0.01)	**0.001**
*Step 3*	0.005 (0.002, 0.01)	**0.001**	0.005 (0.002, 0.01)	**<0.001**	0.01 (0.004, 0.01)	**<0.001**
In-Training Examination Percentile						
*ITE 1*	0.002 (0.001, 0.004)	**0.003**	0.002 (0.001, 0.004)	**<0.001**	0.002 (0.001, 0.003)	**0.001**
*ITE 2*	-	-	0.003 (0.001, 0.004)	**<0.001**	0.002 (0.001, 0.004)	**0.002**
*ITE 3*	-	-	-	-	0.003 (0.002, 0.004)	**<0.001**
Referral to CCC for professionalism	−0.15 (−0.26, −0.05)	**0.006**	−0.12 (−0.20, −0.03)	**0.009**	−0.21 (−0.29, −0.13)	**<0.001**

Abbreviations: ACGME – American College of Graduate Medical Education; PGY – Post Graduate Year; IQR- interquartile range; USMLE – United States Medical Licensing Examination; SD – standard deviation; ITE – In-Training Exam; CI – confidence interval; CCC – clinical competency committee.

*Following Bonferroni correction, a P-value threshold of<0.017 was used to determine significance..

Bold indicates *p-*values below the significance threshold.

### Multivariable regression analysis

None of the resident or applicant factors included in the final multivariable regression models (age at starting residency, USMLE Step scores, interview score, rank list position, ITE scores, and referral to CCC) as predictors of resident clinical performance were significantly associated with mean ACGME milestone scores for PGY-1 and PGY-2 (Table 3). For PGY-3 residents, referral to the CCC for professionalism concerns had a significant negative association with mean milestone scores (beta = −0.13 [95% CI −0.22, −0.04], *p* = 0.006).

**Table 3.** Multivariable analysis of predictors of aggregate ACGME Milestone Scores by Post-Graduate Year of Internal Medicine Residency.

Predictor of Average Milestone Score	PGY 1		PGY 2		PGY 3	
	β (95% CI)	*P*-value*	β (95% CI)	*P*-value*	β (95% CI)	*P*-value*
Age at start of residency	−0.013 (−0.03, 0.0002)	0.054	0.001 (−0.01, 0.01)	0.819	−0.003 (−0.01, 0.01)	0.520
Male gender	-	-	-	-	-	-
Interview Score	0.05 (−0.02, 0.12)	0.183	0.02 (−0.04, 0.08)	0.473	0.04 (−0.02, 0.10)	0.182
Rank list position	−0.0001 (−0.001, 0.001)	0.760	−0.0003 (−0.001, 0.0003)	0.264	−0.0001 (−0.001, 0.001)	0.838
USMLE Step Score						
*Step 1*	−0.001 (−0.01, 0.003)	0.543	0.001 (−0.003, 0.004)	0.727	−0.001 (−0.004, 0.10)	0.592
*Step 2*	−0.002 (−0.01, 0.002)	0.319	−0.002 (−0.005, 0.002)	0.366	0.0002 (−0.003, 0.004)	0.887
*Step 3*	0.003 (−0.001, 0.01)	0.191	0.0005 (−0.003, 0.004)	0.795	0.003 (−0.0003, 0.01)	0.070
In Training Examination Percentile						
*ITE 1*	0.002 (−0.0003, 0.004)	0.088	0.001 (−0.001, 0.003)	0.150	0.001 (−0.001, 0.003)	0.574
*ITE 2*	-	-	0.001 (−0.001, 0.004)	0.322	−0.001 (−0.003, 0.001)	0.455
*ITE 3*	-	-	-	-	0.002 (−0.001, 0.004)	0.189
Referral to CCC for professionalism	−0.07 (−0.18, 0.04)	0.232	−0.07 (−0.16, 0.02)	0.148	−0.13 (−0.22, −0.04)	**0.006**

Abbreviations: ACGME – American College of Graduate Medical Education; PGY – Post Graduate Year; IQR- interquartile range; USMLE – United States Medical Licensing Examination; SD – standard deviation; ITE – In-Training Exam; CI – confidence interval; CCC – clinical competency committee.

*Following Bonferroni correction, a P-value threshold of<0.017 was used to determine significance..

Bold indicates *p-*values below the significance threshold.

We performed a post-hoc sensitivity analysis (Appendix) to explore milestone scores for each of the ACGME core competency domains to determine if this association was largely attributable to the Professionalism domain, or if other domains were impacted. This secondary analysis suggests referral to the CCC for professionalism is negatively associated with resident milestone scores across all the core competency domains.

## Discussion

In this study we tested several applicant factors (e.g., Step 1 and Step 2 CK scores, rank list position, age, gender) and resident factors (e.g., Step 3 score, ITE scores, and referral to the CCC for professionalism concerns) as predictors of IM resident performance. Notably, we did not observe consistent associations between applicant or resident factors and resident performance measured by ACGME milestone scores. In our univariable analysis we did observe a negative association with referral to the CCC, as well as weak positive associations between ITE and USMLE scores, with milestone scores. However, following a multivariable analysis only the negative association with referral to the CCC for professionalism among PGY-3 residents was significant.

Several studies have attempted to identify factors within residency applications and faculty or patient evaluations to predict resident performance with mixed results [3,4,10,12,13]. The study by Fine *et al*. concluded there was an overemphasis on Alpha Omega Alpha (AOA) status, medical school reputation, and Step 1 scores, only modestly correlating (*r* = −0.52) to resident performance evaluations [12]. Similarly, Neely *et al*. examined several applicant factors to derive a weighted algorithm to predict resident performance [13]. In contrast to the conclusions of Fine *et al*., the Neely algorithm relies heavily on medical school quality and Step 1 scores. The work by Sharma *et al*. suggested USMLE Step 2 CK was the best predictor of residency performance when measured by a multimodal ambulatory care evaluation [4]. These conflicting results are difficult to generalize as each of these studies relied on non-standardized assessments of resident performance prior to implementation of the ACGME milestones. Although the ACGME milestones may not be a perfect measure of performance, they stand as the most widely used metric of resident performance available [14–18]. Recently, Golden *et al*. examined the associations between applicant factors and ACGME milestones as a reflection of resident performance limited to intern year [10]. They concluded ‘most traditional metrics used in residency selection were not associated with early performance on ACGME milestones during internal medicine residency.’

Our study has several strengths and builds on previous efforts to predict IM resident performance and warrants further discussion. First, we utilized a widely used metric to assess resident performance, the ACGME milestones [19]. This enhances the external validity and generalizability of our findings across IM programs compared to older studies that relied on institution-specific evaluation systems [4,12,13]. Next, we included milestone evaluations from multiple types of practice settings (e.g., ambulatory care clinics, general medicine wards, subspecialty inpatient wards, subspecialty clinics, intensive care units, etc.) encompassing every clinical site residents rotate through in our program. This provides a more comprehensive assessment of resident performance compared to a single practice setting (i.e., ambulatory care clinic) as in the Sharma *et al*. study [4]. Furthermore, we examined resident performance longitudinally across all 3 years of IM training for categorical residents entering our residency program, as opposed to restricting our analysis to the intern year alone. Longitudinal assessment could avoid missing discrepancies in performance that may arise as residents undergo shifting expectations over the course of their training. Based on these study design strengths, we conclude the applicant factors used (e.g., Step 1, Step 2 CK, rank list position, interview score) to guide the resident selection process do not predict resident performance based on ACGME milestone evaluation scores.

Several associations between resident factors (e.g., Step 3 and ITE scores) and resident milestone scores were identified in our analysis, all with small effect sizes (Table 2) and are of uncertain significance. However, referral to the CCC for professionalism issues demonstrated the largest effect size and was negatively associated with resident milestone scores during PGY-3. We speculate the inverse relationship between referral to the CCC and resident milestone scores may be related to underlying professionalism issues negatively affecting a resident’s overall performance. Professionalism is an important characteristic to measure as previous reports suggest individuals with unprofessional behavior during medical school and residency have higher rates of disciplinary action by medical boards during their post-training careers [20,21]. Furthermore, Dupras *et al*. reported that ‘residents in difficulty’ with professionalism concerns often had deficiencies in multiple competencies [22]. While unprofessional behavior seems like a characteristic that would be easy to identify during the residency selection process, only one-third of program directors could retrospectively identify residents at risk for poor performance based on application materials [22]. We do not have a solution that will assist program directors with this dilemma, though some potential tools already exist in the residency application. One of these tools is the professionalism section of the Medical Student Performance Evaluation (MSPE), which was identified as the most important section of the MSPE by program directors and selection committees across all specialties in a recent study by Bird *et al*. (though there was remarkable distrust of the MSPE by program directors) [23]. Professionalism concerns may also hint to underlying factors such as burnout or mental health disorders that could broadly affect performance. In summary, we propose referral to the CCC for professionalism may be a predictor of resident performance and should alert program directors to potential professionalism issues or underlying resident factors that negatively impact resident milestone scores across all core competencies.

This study is not without limitations. The single center, retrospective design is a key study limitation and may limit the generalizability of our findings. Additionally, by only including residents who matched in our IM program, we recognize that selection bias may limit our ability to detect factors that predict performance. Another limitation is that only one specialty (IM) was included so our conclusions may not apply to other specialties. The main outcome measure used in our study, mean ACGME milestone score, has been questioned and may not accurately reflect a resident’s global performance [15,24], although milestones remain the most widely studied measure to date we concede that aggregating milestone scores may not fully capture resident performance over time and assessors may vary in how they assign milestone scores. It is possible other unmeasured factors that were not studied may be more predictive of resident performance (e.g., personality traits, mental/physical illness during residency, resident wellness, medical school performance, participation in sports teams, etc.) [7]. The use of rank list is another limitation as this is a combination of various factors both subjective and objective. Anecdotally, the generation of rank lists occupies a large portion of program directors’ time. Rank list has been shown to correlate with higher ACGME milestone scores in a univariate analysis in one study [25]. Our results did not demonstrate a significant correlation with higher milestone scores. These conflicting findings question the utility of the rank list in predicting resident success. Lastly, the Bonferroni correction that was used is a more conservative approach to adjust for family-wise error rate when conducting multiple tests. It is possible that the type 2 error rate was increased while trying to improve the type 1 error rate [11].

Future studies are needed to test these metrics in other specialties and verify our findings within a broader population, particularly our findings on the impact of referral to the CCC. Further studies should explore additional factors including research in medical school, volunteer work, and the professionalism section of the MSPE. Finally, while milestones represent a fair measure of assessment, continuing to evaluate their effectiveness as a metric of resident performance as compared to other measures remains important.

## Conclusion

In our study, common residency selection factors did not predict IM resident milestone evaluation scores. Referral to the CCC for professionalism was correlated with worse resident milestone scores across all domains during PGY-3, suggesting professionalism issues correlate with clinical performance.

## Appendix

Sensitivity Analysis of ACGME Milestone Core Competencies for Resident Physicians Referred to the Clinical Competency Committee.

**Table:**

ACGME Milestone Core Competency	PGY 1		PGY 2		PGY 3	
	β (95% CI)	*P*-value*	β (95% CI)	*P*-value*	β (95% CI)	*P*-value*
**Medical Knowledge**						
*Referral to CCC*	−0.117 (−0.231, −0.003)	0.044	−0.084 (−0.165, −0.004)	0.039	−0.198 (−0.281, −0.114)	**<0.001**
**Professionalism**						
*Referral to CCC*	−0.123 (−0.260, −0.013)	0.077	−0.072 (−0.177, 0.034)	0.181	−0.157 (−0.252, −0.062)	**0.001**
**Patient Care and Procedural Skills**						
*Referral to CCC*	−0.168 (−0.287, −0.048)	**0.007**	−0.126 (−0.222, −0.030)	**0.011**	−0.221 (−0.309, −0.133)	**<0.001**
**Interpersonal and Communication Skills**						
*Referral to CCC*	−0.166 (−0.273, −0.058)	**0.003**	−0.118 (−0.209, −0.0.27)	**0.012**	−0.167 (−0.260, −0.077)	**<0.001**
**Systems-Based Practice**						
*Referral to CCC*	−0.180 (−0.295, −0.064)	**0.003**	−0.180 (−0.304, −0.056)	**0.005**	−0.274 (−0.378, −0.170)	**<0.001**
**Practice-Based Learning and Improvement**						
*Referral to CCC*	−0.161 (−0.292, −0.030)	**0.016**	−0.274 (−0.402, −0.145)	**<0.001**	−0.261 (−0.392, −0.160)	**<0.001**

Abbreviations: ACGME – American College of Graduate Medical Education; PGY – Post Graduate Year; IQR- interquartile range; USMLE – United States Medical Licensing Examination; SD – standard deviation; ITE – In-Training Exam; CI – confidence interval; CCC – clinical competency committee.

**Following Bonferroni correction, a P-value threshold of<0.017 was used to determine significance*..

Bold indicates *p-*values below the significance threshold.
